# Crossed beam energy transfer between optically smoothed laser beams in inhomogeneous plasmas

**DOI:** 10.1098/rsta.2020.0038

**Published:** 2020-10-12

**Authors:** Stefan Hüller, Gaurav Raj, Mufei Luo, Wojciech Rozmus, Denis Pesme

**Affiliations:** 1 Centre de Physique Théorique (CPHT), CNRS, Ecole Polytechnique, IP Paris, 91128 Palaiseau, France; 2 Key Laboratory for Laser Plasmas (MoE), Department of Physics and Astronomy, Jiao Tong University, Shanghai, China; 3 Theoretical Physics Institute, Department of Physics, University of Alberta, Edmonton, Alberta, Canada

**Keywords:** laser–plasma instabilities, plasma waves, lasers smoothing techniques

## Abstract

Crossed beam energy transfer, CBET, in high-intensity laser–plasma interaction is investigated for the case of optically smoothed laser beams. In the two approaches to laser-driven inertial confinement fusion experiments, the direct-drive and the indirect-drive, CBET is of great importance because it governs the coupling of laser energy to the plasma. We use the two-dimensional wave-coupling code Harmony to simulate the transfer between two laser beams with speckle structure that overlap in a plasma with an inhomogeneous flow profile. We compare the CBET dynamics for laser beams with spatial incoherence and with spatio-temporal incoherence; in particular we apply the smoothing techniques using random phase plates (RPPs) and smoothing by spectral dispersion (SSD), respectively. It is found that for laser beams (wavelength *λ*_0_) with intensities (*I*_*L*_) above *I*_*L*_ ∼ 2 × 10^15^ W cm^−2^(*λ*_0_/0.35 µm)^−2^(*T*_*e*_/keV), both the so-called plasma-induced smoothing as well as self-focusing in intense laser speckles induce temporal incoherence; the latter affects the CBET and the angular distribution of the light transmitted behind the zone of beam overlap. For RPP-smoothed incident beams, the resulting band width of the transmitted light can already be of the same order as the effective band width of the SSD available at major laser facilities. We examine the conditions when spatio-temporal smoothing techniques become efficient for CBET.

This article is part of a discussion meeting issue ‘Prospects for high gain inertial fusion energy (part 1)’.

## 1. Introduction

Crossed beam energy transfer, CBET, is a process that arises when two or more propagating laser beams intersect in an active medium like an underdense plasma [1–3]. Crossing laser beams induce periodic density perturbations via the ponderomotive force of the overlapping fields acting on the electrons. These density perturbations are periodic in the direction of the difference wavevector between the laser beams. CBET leads to a net transfer between the beam energy, if the induced plasma response is in resonance with the ponderomotive force. The interaction between the laser beams and the induced density perturbation can become a resonant wave coupling process either when the laser waves have different frequencies or when the laser waves have equal frequencies in case of sonic flow inside a plasma flow profile. The understanding of the transfer between intense laser beams is of great importance for inertial confinement fusion (ICF), in order to control the laser–plasma coupling both for the direct drive and the indirect drive schemes. Target design studies can produce highly optimized laser–plasma coupling scenarios for both schemes. However, the quality of the target design is strongly affected by changes in the laser beams resulting from uncontrolled CBET, in particular when the on-target energy deposition does not correspond to what is assumed in the design study.

CBET belongs to the numerous laser–plasma interaction processes that are difficult to describe within computational target design. The reasons for these difficulties are spatial and temporal scale separations between large scale hydrodynamical modelling and the rapid and small-scale evolution of wave coupling processes leading to CBET.

In this article we will study the impact of currently available optical laser beam smoothing techniques on the energy transfer between crossing beams. On the fine scale of laser wavelength (*λ*_0_) the intensity profiles of so-called smoothed laser beams have a speckle structure with known statistical distribution *f*(*I*_sp_) of the speckle peak intensity *I*_sp_ [4]. Statistical distribution of speckles prevents large intensity variation within laser beam and this mitigates the onset of self-focusing and other laser–plasma interaction instabilities. These nonlinear processes occur only in a small percentage of high intensity speckles of the randomized laser beam, which should limit their effect on the overall energy coupling to the plasma.

We will focus here on CBET and laser–plasma instabilities in the presence of spatio-temporal smoothing. In particular we compare CBET between crossing beams with spatial smoothing using random phase plates (RPPs) [4] and beams that have spatio-temporal smoothing by means of the smoothing by spectral dispersion (SSD) technique [5–8]. In SSD the temporal smoothing, in addition to the spatial smoothing, is introduced via a frequency modulation and an amplitude modulation in the phase of the light field. Similarly to the phase plate method, SSD also produces in the focal region of the beam an intensity pattern that consists of numerous speckles. However, and in contrast to the case of RPPs, these speckles continuously move around [9]. The latter motion is supposed to reduce the coherence of parametric instability processes which are undesired effects in the context of laser fusion. SSD is the spatio-temporal smoothing technique which is currently available and used at all major laser facilities.

In our recent work [10,11] we have investigated the role of laser speckles (or laser hot spots) on CBET of two crossing spatially smoothed laser beams. We have shown that taking into account speckles of RPP beams changes considerably both (i) the energy transfer and (ii) the angular spread of transmitted light (beyond the spread defined by the optics of two crossed beam) as compared to the standard description of CBET that ignores speckle structure [12–16].

Recently, studies taking into account the speckle structure of beams in the context of CBET in laser fusion schemes have been performed both for direct drive [17,18] and indirect drive [19,20] schemes, partly relying on the linear plasma response [18,19].

Our study is focused on the case of beam crossing under a relatively small angle of the order 20^°^, and on the nonlinear response of the plasma to the ponderomotive force that acts on the plasma fluid due to the overlapping beams and the speckle structure inherent to each beam. In particular, we have found that nonlinear processes produced by the intense speckles in the flowing plasma such as beam bending [21–23] and plasma-induced smoothing [24–26] develop already at laser beam intensities, *I*_*L*_ ∼ 2 × 10^15^ W cm^−2^(*λ*_0_/0.35 µm)^−2^(*T*_*e*_/keV) (with *λ*_0_ denoting the laser wavelength and *T*_*e*_ the electron temperature). This is particularly relevant for plasmas with sub-sonic flow, close to the sonic layer, for which it has been shown that the threshold for self-focusing is reduced as compared to stationary plasmas with zero flow velocity [27,28]. Another important result of these studies is the onset of temporal incoherence seen in the light beams transmitted behind the cross-interaction region. The coherence times seen in the simulation results [11] correspond to a bandwidth range on the order of 50–100 GHz, being comparable to the magnitudes of bandwidth that are currently produced by temporal smoothing techniques such as SSD at major facilities.

In this article we present results of a study, based on numerical simulations with our paraxial wave interaction code Harmony [29]. We will consider the crossing of two laser beams each one generated with the SSD spatio-temporal smoothing technique. We will study the sensitivity of CBET on the modulation frequency and on the phase amplitude of the SSD technique, in order to understand the effect of temporal smoothing on the net energy transfer and the angular spread of the transmitted beams. We will build on the previous results (see refs [10,11]) that demonstrated plasma-induced smoothing with coherence times of 1–3 ps for RPP beams (for laser light with a wavelength of *λ*_0_ = 0.35 µm).

## 2. Geometry of interaction and numerical modelling

We model the interaction between two laser beams crossing at the angle ϑ in a plasma with inhomogeneous flow. The chosen configuration corresponds to two ‘s’-polarized beams crossing at a relatively small angle ϑ, having the common wavevector component along the positive *x* direction. Such a configuration is relevant to the geometry of many crossing beams at laser entrance holes in the indirect drive ICF experiments. Owing to the superposition of the electromagnetic fields and the resulting ponderomotive force, ion acoustic density perturbations will be induced with a wavevector along the *y* direction.

**Figure 1.**
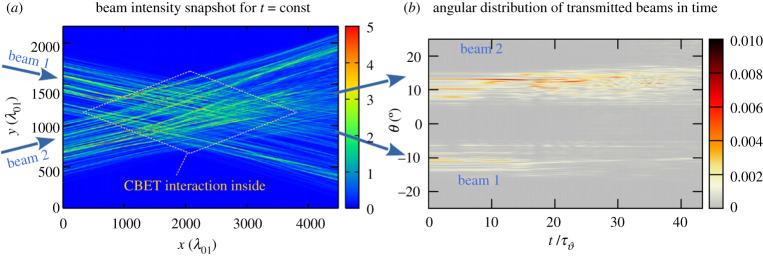
(*a*) Intensity contour map snapshot taken at $t=43.4\tau_{\vartheta }$ (in units of $\tau_{\vartheta }={\left(2k_{1}c_{s}\sin\vartheta /2\right)}^{-1}$), showing the superposition of the two RPP beams for the case when both incident laser beams have an intensity *I*_2_ = *I*_1_ = 3*I*_0_. Contour values correspond to light intensity with respect to the incident beam value, here 3*I*_0_. The inserted rhombus (in yellow) indicates the region of beam overlap where CBET takes place. (*b*) Angular distribution, as a function of time, of the light beams transmitted beyond the interaction region. Temporal incoherence in the transmitted light arises for times $t>17\tau_{\vartheta }$ with a typical coherence time $\tau_{c}∼2.6\text{–}5.2\tau_{\vartheta }$ for this case. (Online version in colour.)

The geometry of the beam overlap region is illustrated in figure 1*a*. Here, the inhomogeneous plasma with a flow is characterized by the profile, *v*_*p*,*y*_(*y*)***e***_*y*_, having the dominating flow direction along the *y*-axis. In the configuration of figure 1*a*, plasma flow is defined by the inhomogeneous flow profile *v*_*p*,*y*_(*y*)/*c*_*s*_ = (*y* − *L*_*y*_/2 + *L*_*v*_)/*L*_*v*_ with *L*_*v*_ = 200*λ*_0_ in the simulations. The flow is sonic at *y*/*λ*_0_ = *L*_*y*_/2 = 1100, and is sub- (super)-sonic for *y*/*λ*_0_ < ( > )1100, respectively. This geometry corresponds to strong coupling between beams crossing at a small angle.

For two ‘s’-polarized beams, with wave vectors and frequencies (***k***_1_, *ω*_1_) and (***k***_2_, *ω*_2_), the matching conditions for resonance correspond to stimulated Brillouin forward scattering (SBFS); the conditions are satisfied when *v*_*p*,*y*_/*c*_*s*_ = (*ω*_1_ − *ω*_2_ − *σω*_*s*_)/(*c*_*s*_*k*_*s*_), with *σ* denoting the sign of *ω*_1_ − *ω*_2_ − (***k***_1_ − ***k***_2_) · ***v***_*p*_, and where $c_{s}\equiv {\left[(c_{se}^{2}/(1+k_{s}^{2}\lambda_{\text{De}}^{2})+3v_{i}^{2}\right]}^{1/2}$ is the IAW velocity, with *c*_*se*_ ≡ (*ZT*_*e*_/*m*_*i*_)^1/2^; here *T*_*e*_ is the electron temperature, *λ*_De_ the Debye length, *v*_*i*_ the ion thermal velocity, *m*_*i*_ and *Z* are the ion mass and charge number, respectively.

To describe the propagation of the light beams in Harmony, we model the laser electric field via complex field envelopes and solve the paraxial wave equation,

2.1 $$[2i\omega_{1}(\partial_{t}+v_{gx}\partial_{x})+c^{2}{\left(\nabla^{2}\right)}_{⊥}]a(x,t)=\omega_{p}^{2}\frac{\delta n}{n_{e}}a(x,t),$$

in which *ω*_1_ is the laser frequency of beam 1 and *a*(***x***, *t*) the normalized laser field envelope. *ω*_*p*_ = (*n*_*e*_e^2^/ε_0_*m*_*e*_)^1/2^ is the electron plasma frequency; $n_{c}=\varepsilon_{0}m_{e}\omega_{0}^{2}/{\text{e}}^{2}$ denotes the critical density, *m*_*e*_ and *e* being the plasma electron mass and charge respectively, *δn* = (*n* − *n*_*e*_) is the density perturbation about the equilibrium density *n*_*e*_. In equation (2.1) the field envelope, *a*(***x***, *t*) ≡ *a*_1_(***x***, *t*)exp{*i****k***_1,*y*_ · ***y***} + *a*_2_(***x***, *t*)exp{ − *i****k***_1,*y*_ · ***y***} is composed of two superposed fields, *a*_1_, *a*_2_, corresponding to the two beams incident at the angles ±ϑ/2 to the *x*-axis; $k_{∥}$ and ***k***_*j*,*y*_ are the parallel and transverse components of the wave vectors, respectively, with $|k_{∥}|=|k_{j}|\cos(\vartheta /2)$, $|k_{j,y}|=|k_{j}|\sin(\vartheta /2)$ and $|k_{j}|={\left(\omega_{j}^{2}-\omega_{p}^{2}\right)}^{1/2}/c\;(j=1,2)$. The group velocity in equation (2.1) is given by $v_{gx}=c^{2}k_{∥}/\omega_{1}$. The total field is defined as

2.2 $$E(x,t)=\frac{\hat{E}}{2}\;{\text{e}}^{-i\omega_{1}t+ik_{∥}\cdot x}[a_{01}\;{\text{e}}^{ik_{1,y}\cdot y}+a_{02}\;{\text{e}}^{-i(\omega_{2}-\omega_{1})t+ik_{2,y}\cdot y}]+cc,$$

in which $\hat{E}$ is the dimensional field strength, and *ω*_1_, *ω*_2_ are the frequencies of laser beam 1 and 2. The envelope approximation holds for |∂_*t*_*a*_*j*_| ≪ |*ω*_*j*_*a*_*j*_| and $|\partial_{x}a_{j}|\ll |k_{∥}a_{j}|\;(j=1,2)$.

For the cases we consider in this study, we assume always that both beams have the same frequency *ω*_2_ = *ω*_1_, which is consistent with the CBET resonance around sonic flow (at *y* ≃ *L*_*y*_/2 ≃ 1100*λ*_0_). Furthermore we restrict our analysis to the case when both beams have initially the same average intensity, *I*_0_ = *I*_2,in_ = *I*_1,in_ with $I_{1,2,\text{in}}=(1/4)\epsilon_{0}c{\hat{E}}^{2}\langle |a_{1,2}{|}^{2}\rangle$, where the averaging denoted by 〈…〉, means for spatially smoothed beams the average across the beam (perpendicular to the propagation axis), and for beams with spatio-temporal smoothing 〈…〉 indicates averaging across the beam and along the axis for a distance comparable to the propagation length, ∼*c*/*ν*_SSD_, with *ν*_SSD_ denoting the effective band width of the temporal smoothing technique.

### (a) Optical smoothing: RPP and SSD

In our simulations we compare spatially smoothed beams, generated by RPPs, with spatio-temporally smoothed beams generated by the SSD technique. Spatio-temporal smoothing is achieved by introducing phases *ϕ*_*j*,*i*_ in the near field (near lens) of the two light beams *a*_*j*_, via

2.3 $$a_{j}(y)={\text{e}}^{ik_{j,y}\cdot y}\sum_{k_{i}=-△k}^{△k}|{\hat{a}}_{j,i}|{\text{e}}^{ik_{i}y+i\phi_{j,i}}\;(j=1,2),$$

where the index *i* numbers the *i*th phase plate of *N*_PP_ elements in total, having the amplitude ${\hat{a}}_{j,i}$ and the wavenumber *k*_*i*_ with respect to the central beam wavenumber component *k*_*j*,*y*_. The wavenumber spread △k is related to the angular width of the beams; it is determined by the so-called ‘focusing *f*-number’, namely $△k=|k_{1}|/\sqrt{1+4f^{2}}$ ≃|*k*_1_|/(2 *f*) [30].

To generate spatial smoothing via RPP beams [30], the phases introduced in the fields *ϕ*_*j*,*i*_ = *ϕ*_*j*,*i*,RPP_ are random values between 0 and *π* for each different phase plate element. The spacing between *N*_PP_ elements follows $k_{i+1}-k_{i}=2△k/N_{\text{PP}}$. The field amplitudes ${\hat{a}}_{j,i}$ of the phase plate elements *i* = 1… *N*_PP_ are considered to be constant in the interval $k_{j,y}-△k\leq k_{y}\leq k_{j,y}+△k$. With respect to the angular width of the beam, it has to be ensured that both beams (*j* = 1, 2) are well separate in the near field representation. For this reason, the angle between their initial directions of propagation has to be greater than the angular width of each beam, yielding the condition $△k<|k_{1,y}|$. The field in the interaction region where the beams cross is computed from the expression of equation (2.3) via a Fourier transform and a paraxial propagator.

For the case of spatio-temporal smoothing with SSD, an additional phase *ϕ*_*j*,*i*,SSD_ is introduced in the near fields, equation (2.3) of the beams, following a deterministic expression often written in the form [7] *ϕ*_*j*,*i*,SSD_ = 3*δ*sin [2*πν*_mod_(*t* + *ξ*_*x*,*i*_*x* + *ξ*_*y*,*i*_*y*)]. The phase *ϕ*_*j*,*i*,SSD_ depends on the frequency modulation *ν*_mod_ and the amplitude of modulations, the phase depth *δ*. In the case of a tripled laser frequency, for the wavelength *λ*_0_ = 0.35 µm as considered in our study, the effective depth assumes a three times higher value, namely 3*δ* in *ϕ*_*j*,*i*,SSD_. The spatial phase variation *ξ*_*x*,*i*_*x* and *ξ*_*y*,*i*_*y* terms stand for ‘longitudinal’ and ‘transvere’ smoothing, respectively, both considered for MJ-class lasers. We concentrate here on transverse smoothing only, most commonly used because of its better smoothing efficiency [31]. The spatial dependence in the phase, *ϕ*_*j*,*i*,SSD_, of transverse SSD is related to the angular aperture via $2\pi \nu_{\text{mod}}\xi_{y,i}y\to 2\pi N_{\text{cc}}k_{i}/(2△k)$, with Δ*k* and discrete values *k*_*i*_ from the phase plates, via the so-called number of ‘colour cycles’ *N*_cc_. The total phase *ϕ*_*j*,*i*_ in equation (2.3) for spatio-temporal smoothing with SSD, for the case of frequency-tripled lasers, can then be written in the form [6,9,32]

2.4 $$\phi_{j,i}=\phi_{j,i,\mathrm{RPP}}+\phi_{j,i,\text{SSD}}=\phi_{j,i,\mathrm{RPP}}+3\delta \sin[2\pi \nu_{\text{mod}}t+\pi N_{\text{cc}}(k_{i}/△k)],$$

in which, *k*_*i*_ assumes again *N*_PP_ discrete values in the interval $-△k\leq k_{i}\leq △k$. In the majority of our simulations we use a single colour cycle *N*_cc_ = 1, except where indicated differently. Using expansion of the phase factor $e^{i3\delta \sin[2\pi \nu_{\text{mod}}t]}$ into a sum of Bessel functions *J*_*m*_(*δ*), it can easily be shown that the Bessel modes *m* = *ν*/*ν*_mod_ of the order −3*δ* < *m* < 3*δ* have to be retained, resulting in the effective temporal bandwidth of SSD beam 3*ν*_SSD_ ∼ 3(2*δν*_mod_). Typical values for modulation frequencies reported for major laser facilities are *ν*_mod_ = 14.25 GHz at the French LMJ [8], *ν*_mod_ = 3.3 GHz with *δ* = 6.15 for transverse and 10.4 GHz with *δ* = 14.3 for longitudinal SSD at LLE Rochester [7], and *ν*_mod_ = 17 GHz at NIF [6].

### (b) Plasma dynamics

The interaction with the plasma is modelled in the isothermal approximation via the system of the standard continuity and momentum fluid equations for plasma density and velocity, respectively,

2.5*a* $$\partial_{t}n+\nabla \cdot (n\upsilon )=0$$

and

2.5*b* $$\partial_{t}\upsilon +(\upsilon \cdot \nabla )\upsilon +c_{s}^{2}\frac{\nabla n}{n}+2\nu_{s}\upsilon =-c_{se}^{2}\nabla U/T_{e}.$$

We assume for the damping operator *ν*_*s*_ a linear wavenumber dependence accounting for Landau damping; *nν*_*s*_**υ** is computed in Fourier space with $\nu_{s}(k_{s})=\hat{\nu }\;\omega_{s}(k_{s})$. The ponderomotive potential *U* in the source term of the momentum equation is given by $U=T_{e}\Gamma \;\nabla |a(x,t){|}^{2}$ with the coupling coefficient $\Gamma =v_{\text{osc}}^{2}/(2v_{\text{th}}^{2})$ or, in practical units, $\Gamma ≃0.09I_{L}\lambda_{0}^{2}\;(10^{15}\;\text{W}\;\mu {\text{m}}^{2}/{\text{cm}}^{2})/T_{e}(\text{keV})$. The coupling constant involves the thermal velocity *v*_th_ = (*T*_*e*_/*m*_*e*_)^1/2^ and the electron quiver velocity $v_{\text{osc}}=e\hat{E}/(m_{e}\omega )$ of the field strength $\hat{E}$ (to which *a*_1_ and *a*_2_ are normalized). As we have pointed out in ref. [11], the ponderomotive force resulting from the two beams crossing under a small angle can be subdivided into two major components $\nabla U=\nabla U_{\text{cross}}+\nabla U_{\text{self}}$, namely $\nabla U_{\text{cross}}/T_{e}≃\Gamma \;\nabla a_{1}a_{2}^{*}\;{\text{e}}^{2i|k_{1}|y\sin(\vartheta /2)}+cc.$ and $\nabla U_{\text{self}}/T_{e}≃\Gamma \;\nabla (|a_{1}{|}^{2}+|a_{2}{|}^{2})$. The ‘cross’ term arises for wave-coupling interaction between crossing beams even in absence of beam speckle structure; it is therefore usually retained for the modelling of CBET for unstructured beams or plane waves [12–16,19]. The term denoted with ‘self’, standing for beam self-interaction, however, accounts for nonlinear effects arising inside the beams, such as beam self-focusing and plasma-induced smoothing [24–26] due to stimulated Brillouin forward scattering, as well as beam bending [21–23] in presence of sonic plasma flow.

As it has been demonstrated in refs [10,11], both terms have to be taken into account for a correct description of CBET with optically smoothed laser beams in flowing plasmas.

### (c) Modelling crossed beam energy transfer

The theory describing the crossing of two structure-less beams has been developed in the work by McKinstrie [33] by describing the coupling in a geometry based on oblique, non-orthogonal coordinates *η* and *ξ*, *x* = *η****e***_*x*_ · ***e***_*η*_ + *ξ****e***_*x*_ · ***e***_*ξ*_ and *y* = *η****e***_*y*_ · ***e***_*η*_ + *ξ****e***_*y*_ · ***e***_*ξ*_ with $e_{y}\cdot e_{\eta }=-\sin(\phi -\vartheta /2)$ and $e_{x}\cdot e_{\xi }=-\sin(\phi +\vartheta /2)$. With the choice *ϕ* = 0 for the angle between the plasma flow ***v***_*p*_ and the IAW vector ***k***_*s*_ = ***k***_1_ − ***k***_2_ one obtains $e_{y}\cdot e_{\eta }=\sin(\vartheta /2)$ and $e_{x}\cdot e_{\xi }=-\sin(\vartheta /2)$ in two-dimensional geometry. By neglecting the diffraction term of the paraxial propagation, the coupling can be described by the system of partial differential equations [15] for the beam amplitudes *a*_1_ and *a*_2_

2.6 $$\begin{array}{r} \begin{array}{rl} & \partial_{\eta }\partial_{\xi }|a_{1}{|}^{2}=-2\partial_{\eta }(\beta (\xi ,\eta )\;|a_{1}{|}^{2}\;|a_{2}{|}^{2})\\ \;\text{and}\; & \partial_{\xi }\partial_{\eta }|a_{2}{|}^{2}=2\partial_{\xi }(\beta (\xi ,\eta )\;|a_{1}{|}^{2}\;|a_{2}{|}^{2}). \end{array} \end{array}$$

The function *β*(*ξ*, *η*) accounts for the geometry of the crossing zone, having a rhombus-like shape, and is given by $\beta (\xi ,\eta )=(\gamma_{0}^{2}/\nu_{s}c_{s})/[1+{\left(Q_{0}-Q(\xi ,\eta )\right)}^{2}k_{s}^{2}c_{s}^{2}/\nu_{s}^{2}]$, with $\gamma_{0}^{2}\equiv (n_{e}/n_{c})(\omega_{1}/\omega_{s})k_{s}^{2}c_{s}^{2}v_{\text{osc}}^{2}/(4v_{\text{th}}^{2})=(n_{e}/n_{c})(\omega_{1}/\omega_{s})k_{s}^{2}c_{s}^{2}\Gamma /2$, where *γ*_0_ denotes the SBS standard temporal growth rate. The auxiliary functions *Q*_0_ and *Q*(*ξ*, *η*) are given by *Q*_0_ = −1 + (*ω*_1_ − *ω*_2_)/(*k*_*s*_ *c*_*s*_) and, for *ϕ* = 0, $Q=|(v_{p}(L_{y}/2)/c_{s})+2(y-L_{y}/2)\sin(\vartheta /2)/L_{v}|$ with *v*_*p*,*y*_(*y*)/*c*_*s*_ = (*y* − *L*_*y*_/2 + *L*_*v*_)/*L*_*v*_. This allows one to identify a gain coefficient for SBS-induced CBET given by [11,15]

2.7 $$G\equiv \frac{2\gamma_{0}^{2}}{\nu_{s}c_{s}}\min\left\{\frac{D}{2\sin\vartheta },L_{\text{inh}}\right\},$$

by considering the domain of integration as defined by the beam width *D*; note that $\gamma_{0}^{2}=\gamma_{\pi }^{2}\sin(\vartheta /2)$ with *γ*_*π*_ standing for the SBS growth rate for backscattering. The length $L_{\text{inh}}=\pi (L_{v}\nu_{s}/\omega_{s})/\sin(\vartheta /2)$ is related to inhomogeneous flow, which we consider here together with equal frequencies for both beams, *ω*_1_ = *ω*_2_.

(a) For the case $D/(2\sin\vartheta )>L_{\text{inh}}$, the exchange between the beams covers only a restricted zone inside the rhombus-shaped region of beam overlap; the resulting gain $G\to G_{\text{max}}=2\pi \gamma_{0}^{2}L_{v}/[c_{s}\omega_{s}\sin(\vartheta /2)]$ is independent of the damping. The value of *G*_max_, given in practical units by *G*_max_ ≃ 0.75 (*I*_*L*_/10^15^ W cm^−2^) (*λ*_0_/0.35 µm)^2^ (*T*_*e*_/3 keV)^−1^ (*n*/0.1*n*_*c*_) (*L*_*v*_/200*λ*_0_), indicates in reality an upper bound for *G*.
(b) For the opposite case $L_{\text{inh}}>D/(2\sin\vartheta )$, the exchange between the beams takes place over the entire region of overlap.

In most of our simulations presented in the following, case (a) applies.

The principal observable of CBET is the transfer rate between the beams, relating the transmitted power of the amplified beam to its incoming power; it is defined as *T* ≡ *P*_out_/*P*_in_. We consider beam 2 as the amplified beam to which *T* applies.

The asymptotic transfer rate [15,33] for this beam can be expressed, as a function of the gain *G* by evaluating the coupling considering the incident beam 1 as the pump beam, $\Gamma ∼\gamma_{0}^{2}∼I_{1,\mathrm{in}}∼|a_{1,\mathrm{in}}{|}^{2}$. It involves also the intensity ratio between both beams $(I_{2}/I_{1})\equiv I_{2,\mathrm{in}}/I_{1,\mathrm{in}}$ and reads $T(G)=1+(I_{1}/I_{2})(1+\log[1+{\text{e}}^{-G(1+I_{2}/I_{1})}-{\text{e}}^{-G}]/G)$; here we also assume that both beams have the same width *D* = *D*_1_ = *D*_2_. For small gain, G≪1, the transfer *T* ≃ 1 + (*G*/2) (1 + *I*_1_/*I*_2_) increases linearly with *G*. For the case of the simulations shown here, we always assume *I*_1_ = *I*_2_. Using the gain value *G* = *G*_max_ in the expression and *T*(*G*) should be considered as an upper bound of the effective gain for crossed beams. In ref. [15], relatively good agreement between the expression *T*(*G*) and simulations is found. In the intensity regime considered in our study, the gain always assumes values *G* >1 such that the regime *T* > 1.5 → 2 is attained even in case of high damping (*ν*_*s*_/*ω*_*s*_ > 0.2), corresponding to the above mentioned case (b) when $D/(2\sin\vartheta )<L_{\text{inh}}$.

## 3. Simulation results

In a preceding study [11] we have performed simulations with crossing laser beams, in which we have compared CBET between unstructured laser beams to CBET between optically smoothed laser beams with the RPP method. As in the current study, we focused on the case of two incident laser beams with equal mean laser intensity $I_{1,\mathrm{in}}=I_{2,\mathrm{in}}$. The results of this study have clearly shown that the self-interaction in crossing laser beams, originating from the ponderomotive force term $\nabla U_{\text{self}}\propto \nabla (|a_{1}{|}^{2}+|a_{2}{|}^{2})$, and acting on the speckle structure in optically smoothed laser beams must not be neglected for plasmas with subsonic to sonic flow [22,27,28].

The criterion for ponderomotive self-focusing in absence of flow, relating the power *P*_sp_ in a speckle with intensity *I*_sp_ to the critical power, which reads in practical units *P*_sp_/*P*_*c*_ = 0.06*η*(*I*_sp_/10^15^ W cm^−2^) (*λ*_0_/0.35 µm)^2^ (*T*_*e*_/3 keV)^−1^ (*f*/8)^2^ (*n*_*e*_/0.1*n*_*c*_), with *η* = 1.23 for 2D geometry, predicts that only the intense speckles would undergo self-focusing (e.g. *I*_sp_ > 5*I*_*L*_ at the average intensity *I*_*L*_ > 3.5 × 10^15^ W cm^−2^ for *λ*_0_ = 0.35 µm and *T*_*e*_ = 3 keV).

However, it has been shown that self-focusing with subsonic flow [22,28] occurs already at lower intensities than expected in absence of flow. According to refs [11,22] there is no longer an onset threshold for self-focusing in the subsonic case. This has consequences for speckles of smoothed light beams that are located in the region of close-to-sonic flow. Beam bending of speckles and enhanced angular spreading contributes to a net transfer into the other beam.

In agreement with this, we find that for a plasma with an inhomogeneous flow profile—as in our simulations—nonlinear effects due to speckle self-focusing and beam bending come into play already for average laser intensities *I*_1_ = *I*_2_ > 0.75 *I*_0_ in terms of the reference intensity *I*_0_ ≃ 0.9 × 10^15^ W cm^−2^ (*λ*_0_/0.35 µm)^−2^ (*T*_*e*_/3 keV).

Furthermore plasma-induced smoothing leads to a non-stationary evolution of the exchange between the beams, which has eventually influence on the CBET rates, on the angular aperture as well as on the temporal coherence of the transmitted light.

In this study we also consider two light beams of equal average intensity *I*_1_ = *I*_2_ which enter into the system with an angle of ±10^°^ i.e. $\vartheta =20^{\circ }$ between them. The time evolution of CBET scales with this angle so that it is useful to introduce the typical time $\tau_{\vartheta }\equiv {\left(2k_{1}c_{s}\sin\vartheta /2\right)}^{-1}$, being the inverse of the sound wave frequency. This time is used to normalize the time axes in our figures.

In figure 1*a* we show the snapshot of the intensity contours of the two crossing RPP beams obtained from two-dimensional simulations with the code Harmony. The transfer between the beams takes place inside the rhombus-shaped area. The plasma is located in the spatial interval 500 < *x*/*λ*_0_ < 4000. The light beam propagates in vacuum for *x*/*λ*_0_ > 4000.

The angular distribution of the transmitted light, as a function of time, shown in figure 1*b*, is determined by taking a Fourier transform of the electromagnetic fields exiting the right-hand-side boundary of the simulation area in figure 1*a*, $a(x=L_{x},y,t)\to \text{FFT}\to {\hat{a}}_{\text{out}}(k_{y},t)$.

Initially both beams have, on average, the same incident intensity and the same angular aperture, ϑ∼1/(2f) where *f* = 6 is the focusing *f*-number. The onset of CBET from beam 1 to beam 2 takes place over a transient interval of $∼17\tau_{\vartheta }$, that corresponds roughly to 3 periods of the acoustic wave induced by CBET (or $2\pi \tau_{\vartheta }∼1.9\;\text{ps}$ for *T*_*e*_ = 3 keV, *λ*_0_ = 0.35 µm and $\vartheta =20^{\circ }$). Generally the transfer rate stabilizes after this transient interval. Based on the angular distribution of the transmitted light, we have determined, using *k*_*y*_ = *k*_1_sin*θ*/2, the transfer rate by computing

3.1 $$\frac{P_{\text{out}}}{P_{\text{in}}}\equiv \frac{\int_{k_{y}>0}|{\hat{a}}_{\text{2, out}}(k,t){|}^{2}\text{d}k}{\int_{k_{y}>0}|{\hat{a}}_{\text{2, in}}(k){|}^{2}\;\text{d}k}.$$

For the RPP case the maximum transfer of *P*_out_/*P*_in_ ≃ 1.75 is reached. As mentioned in §2c, the maximum transfer rate for beams of equal incident intensity is (*P*_out_/*P*_in_)_max_ = 2.

**Figure 2.**
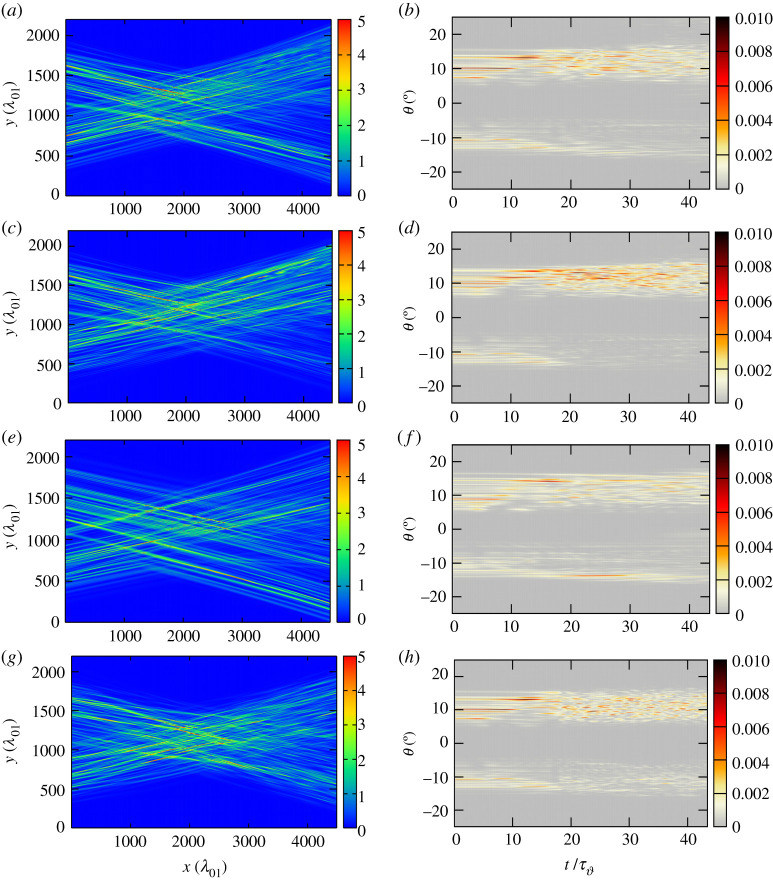
(*a*,*c*,*e*,*g*) As in figure 1, intensity contour map snapshot taken at $t=44\tau_{\vartheta }$ (or 13 ps for *T*_*e*_ = 3 keV); similar to figure 1, here the superposition of two SSD beams is shown, (*a*) SSD with parameters 3*δ* = 6 and *ν*_mod_ = 17 GHz, *N*_cc_ = 1, (*c*) SSD with parameters 3*δ* = 6 and *ν*_mod_ = 17 GHz, *N*_cc_ = 2, (*e*) SSD with *δ* = 18 and *ν*_mod_ = 17 GHz, and (*g*) SSD with 3*δ* = 6 and *ν*_mod_ = 50 GHz, again for the case when both incident laser beams have an intensity *I*_2_ = *I*_1_ = 3*I*_0_. (*b*,*d*,*f* ,*h*) Angular distribution, as a function of time of the light beams transmitted beyond the interaction region. Time is in units $\tau_{\vartheta }={\left(2k_{1}c_{s}\sin\vartheta /2\right)}^{-1}$ (with the conversion $1\;\text{ps}\equiv 3.3\tau_{\vartheta }$ for *T*_*e*_ = 3 keV). The different cases correspond to (*b*) SSD with parameters 3*δ* = 6 and *ν*_mod_ = 17 GHz, *N*_cc_ = 1, (*d*) SSD with 3*δ* = 6 and *ν*_mod_ = 17 GHz, *N*_cc_ = 2, (*f* ) SSD with 3*δ* = 18 and *ν*_mod_ = 17 GHz, *N*_cc_ = 1, and (*h*) SSD with 3*δ* = 6 and *ν*_mod_ = 50 GHz, *N*_cc_ = 1. The temporal incoherence in the transmitted light in cases (*b*) and (*d*) is similar to the RPP case of figure 1; the case (*f* ) has the lowest transfer but stronger angular spread in the transmitted light; the case (*h*) shows considerable reduction in the temporal coherence. (Online version in colour.)

We have performed a series of simulations with SSD for parameters for which we expect that temporal smoothing effects have impact on the CBET transfer. The cases for which we have carried out simulations are case (*a*–*d*), with the parameters 3*δ* = 6 and *ν*_mod_ = 17 GHz, yielding and effective bandwidth 3*ν*_SSD_ ≃ 100 GHz (see §2a): figure 2*a*,*b* displays the sub-case with a single colour cycle *N*_cc_ = 1, and (c–d) the sub-case with two colour cycles *N*_cc_ = 2; the other two cases (both for a single colour cycle *N*_cc_ = 1) correspond to a three times greater effective bandwidth which is obtained by either increasing the phase depth or the modulation frequency by a factor of 3, namely figure 2*e*,*f* with 3*δ* = 18 and *ν*_mod_ = 17 or figure 2*g*,*h* 3*δ* = 6 and *ν*_mod_ = 50 GHz, respectively. The increase of the frequency modulation to such high values may cause in reality difficulties for the laser system, because high bandwidth implies lower conversion efficiencies [8] in the frequency tripling. For this reason, the choice of the case with *ν*_mod_ = 50 GHz should be considered primarily as a study case.

**Figure 3.**
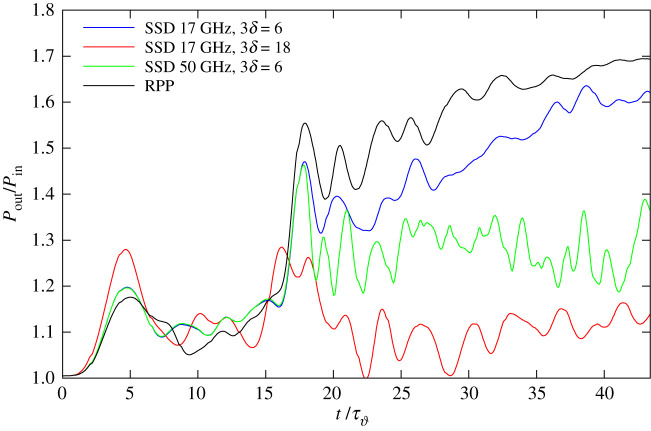
CBET power transfer relating the transmitted power of the two beams with respect to their incident power as a function of time for the cases with incident intensity *I*_1,2_/*I*_0_ = 3. The cases shown correspond to RPPs in blue from figure 1, SSD with parameters 3*δ* = 6 and *ν*_mod_ = 17 GHz in yellow, from figure 2*a*,*b*, SSD with 3*δ* = 18 and *ν*_mod_ = 17 GHz in magenta, figure 2*e*,*f* and SSD with 3*δ* = 6 and *ν*_mod_ = 50 GHz in green, figure 2*g*,*h*. All with *N*_cc_. (Online version in colour.)

The temporal evolution of the transfer rate *P*_out_/*P*_in_ for both beams, as a function of time, for the incident intensity *I*_1,2,in_/*I*_0_ = 3, is shown in figure 3 for three SSD cases and for the RPP case. In equation (3.1), ${\hat{a}}_{\text{2, in}}(k)$ corresponds to the near field of the incident beam, as defined in §2a, which is time-independent for the RPP beams. Note that time averaging has to be performed for temporally incoherent incoming SSD laser beams.

The two simulations with higher effective SSD bandwidth show a clear tendency in reducing the net transfer rate. As far as CBET rates are concerned, the choice of a larger phase depth as in case (*e*–*f* ) with 3*δ* = 18, seems to be the better option to reduce CBET, yielding lower transfer rates, $P_{\text{out}}/P_{\text{in}}≃1.05\text{–}1.14$ than the case (*g*–*h*) with 3*δ* = 6 and *ν*_mod_ = 50 GHz, with still shows considerable transfer *P*_out_/*P*_in_ ≃ 1.4.

As is illustrated in figure 1*b*, the transmitted light from crossing RPP beams shows signatures of temporal incoherence for times *t* >17$\tau_{\vartheta }$, although the incoming light beams have only spatial incoherence. Figure 1*b*, taken from a single RPP realization, shows clearly speckle-structure in time and angle, reminiscent of plasma-induced smoothing. For the shown case, namely with *I*_1,2,in_/*I*_0_ = 3 (with *I*_0_ denoting the reference laser intensity *I*_0_ ≃ 0.9 × 10^15^ W cm^−2^ (*λ*_0_/0.35 µm)^−2^ *T*_*e*_/3 keV), the shortest typical coherence time deduced from these speckles is $\tau_{c}∼2.6\tau_{\vartheta }$. For laser wavelength *λ*_0_ = 0.35 µm and *T*_*e*_ = 3 keV this correspond to 0.8–1 ps. On the basis of this result, it can be expected that spatio-temporal smoothing would not change significantly the CBET between beams by reducing the net transfer, unless a sufficiently large effective bandwidth for SSD, >100 GHz, is introduced to the laser source.

**Figure 4.**
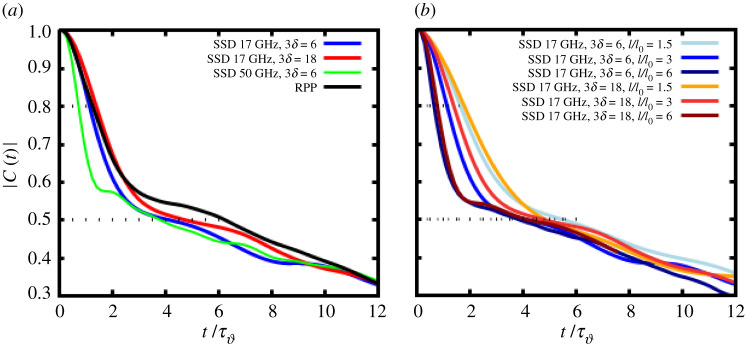
Correlation functions *C*(*t*) of the transmitted light intensity signal of beam 2 in the angular window 10^°^ ± 5^°^ as a function of the time delay *t*; in panel (*a*) the case of RPP is compared to SSD cases with *ν*_mod_ = 17 GHz (blue curve with 3*δ* = 6, red with 3*δ* = 18) and the case *ν*_mod_ = 50 GHz (green curve 3*δ* = 6) for the beam intensity *I*_1_ = *I*_2_ = 3 at the entry; panel (*b*) compares SSD cases with *ν*_mod_ = 17 GHz for three different beam intensity values at the entry, *I*_1_ = *I*_2_ = 1.5, 3, and 6*I*_0_, namely light blue/blue/dark blue curves for 3*δ* = 6, respectively, and yellow/orange/dark red curves for 3*δ* = 18. The time delay axis is in units of $\tau_{\vartheta }={\left(2k_{1}c_{s}\sin\vartheta /2\right)}^{-1}$ and in ps (upper axis) for *λ*_0_ = 0.35 µm wavelength and *T*_*e*_ = 3 keV, via the conversion $\tau_{\vartheta }∼3.3\;\text{ps}\;{\left(T_{e}/3\;\text{keV}\right)}^{1/2}$ (*λ*_0_/0.35 µm)^−1^. (Online version in colour.)

Analysing the intensity contour snapshot and the evolution of angular distribution for the SSD case in figure 2, one can neither see an important change in the CBET transfer rate (*P*_out_/*P*_in_ ≃ 1.5) nor a considerable change in the coherence time ($\tau_{c}≃4.7\text{–}5.2\tau_{\vartheta }$), except that oscillations in the transmitted light become more regular. From the point of view of the angular distribution of the transmitted light, the cases of SSD also differ significantly. While the case with *ν*_mod_ = 50 GHz keeps the beams almost in their original cone, the case with higher phase depth, 3*δ* = 18, has the tendency to produce a greater angular spread of the depleted light beam 1. Also a slightly enhanced angular spread is seen in beam 2. This result is systematic even for other realizations with the same parameters of SSD. It is not surprising that the case with the highest modulation frequency shows the most regular structure in the transmitted beam due to the strongest smoothing features, which is, however, not sufficient to counteract against important CBET net transfer. The correlation times of the transmitted light have been obtained by computing the correlation function from the transmitted intensity signal in angle (*θ*) and in time of light beam 2,

3.2 $$C(t)\equiv \frac{\int_{\vartheta /2-5^{\circ }}^{\vartheta /2+5^{\circ }}\;\text{d}\theta \int \text{d}t^{\prime }|{\hat{a}}_{\text{2, out}}(\theta ,t^{\prime }-t/2){|}^{2}|{\hat{a}}_{\text{2, out}}(\theta ,t^{\prime }+t/2){|}^{2}}{\int_{\vartheta /2-5^{\circ }}^{\vartheta /2+5^{\circ }}\;\text{d}\theta \int \text{d}t^{\prime }\;|{\hat{a}}_{\text{2, out}}(\theta ,t^{\prime }){|}^{4}}$$

taken in the angular interval around $\theta =\vartheta /2\pm 5^{\circ }=10^{\circ }\pm 5^{\circ }$ over the time interval $17<t/\tau_{\vartheta }<44$ (or in real units 5 < *t*/ps < 13.5). The correlation functions displayed in figure 4 show typically features on short time scale and on a longer time scale, not belonging to the same type of decrease.

For this reason, we decided to determine values corresponding to correlations times taking the following criteria: for to the short time behaviour we determine *τ*_*c*,1_ by taking |*C*(*t* = *τ*_*c*,1_/2)| = 4/5 and *τ*_*c*,2_ for the longer time behaviour |*C*(*t* = *τ*_*c*,2_)| = 1/2 (see ref. [26]). The value of *τ*_*c*,1_ obtained in this way mimics a Lorentzian-type decrease $C(t)∼{\left(1+t^{2}/\tau_{c,1}^{2}\right)}^{-1}$. Both values *τ*_*c*,1_ and *τ*_*c*,2_ are reported for each case as possible interval for the correlation time in table 1. It is remarkable, from figure 4*a* that the correlation time values *τ*_*c*,1_ for the case of RPPs and the two 17 GHz SSD cases are very similar for *I*_1,2,in_/*I*_0_ = 3. On the other hand, the correlation functions taken for times corresponding to the period of CBET density perturbations, $t/\tau_{\vartheta }=2\pi$, show lower correlation for both SSD cases with respect to RPPs. For the different intensities of the SSD cases, figure 4*b*, the correlation in the transmitted light diminishes for the short time behaviour.

**Table 1.** Correlation times of the transmitted light deduced from the correlation function *C*(*t*) of the transmitted light intensity signal in the angular window 10^°^ ± 5^°^ of beam 2 for SSD cases with modulation frequency *ν*_mod_ = 17 GHz, and for RPPs at different beam intensities on entry. Values are given in the normalized time units and in ps for the case of *λ* = 0.35 µm and *T*_*e*_ = 3 keV. The time interval indicated, *τ*_*c*,1_ − *τ*_*c*,2_, corresponds to the short time behaviour by taking |*C*(*t* = *τ*_*c*,1_/2)| = 0.8 and |*C*(*t* = *τ*_*c*,2_)| = 0.5 (see ref. [26]).

	*τ*_*c*_ in ${\left(2k_{1}c_{s}\sin\vartheta /2\right)}^{-1}$			*τ*_*c*_ in ps		
*I* =	1.5 *I*_0_	3 *I*_0_	6 *I*_0_	1.5 *I*_0_	3 *I*_0_	6 *I*_0_
RPP		2.6–5.2			0.8–1.6	
SSD 3*δ* = 6	3.6–5.7	2.6–4.1	1.4–3.3	1.1–1.7	0.8–1.3	0.4–1.0
SSD 3*δ* = 18	3.5–4.9	2.9–4.5	1.6-4.1	1.0–1.5	0.9–1.4	0.5–1.2

We have chosen for the IAWs generated by the CBET the damping of *ν*_*s*_/*ω*_*s*_ = 0.1 with $\omega_{s}=\tau_{\vartheta }^{-1}$ in the majority of our simulations. The correlation times deduced from the simulation systematically yield $\tau_{c}<\nu_{s}^{-1}$. For completeness we have also performed selected cases with lower IAW damping of *ν*_*s*_/*ω*_*s*_ = 0.01, 0.03, and higher damping *ν*_*s*_/*ω*_*s*_ = 0.2 and 0.3. The effective SSD bandwidth considered here, $3\delta \;\nu_{\text{mod}}$, is always greater than *ν*_*s*_. For damping higher than *ν*_*s*_/*ω*_*s*_ = 0.2 one would expect from the model in §2c the spatial zone of active CBET coupling should change, and thus the CBET gain value. While CBET-driven IAW density perturbations are not particularly changed by the damping value, density perturbations induced by the ponderomotive force of laser speckles are more pronounced for lower damping. However, similar to what has been seen in ref. [11], neither the higher nor the lower IAW damping values affect considerably the net transfer between the beam with respect to the case with *ν*_*s*_/*ω*_*s*_ = 0.1.

**Figure 5.**
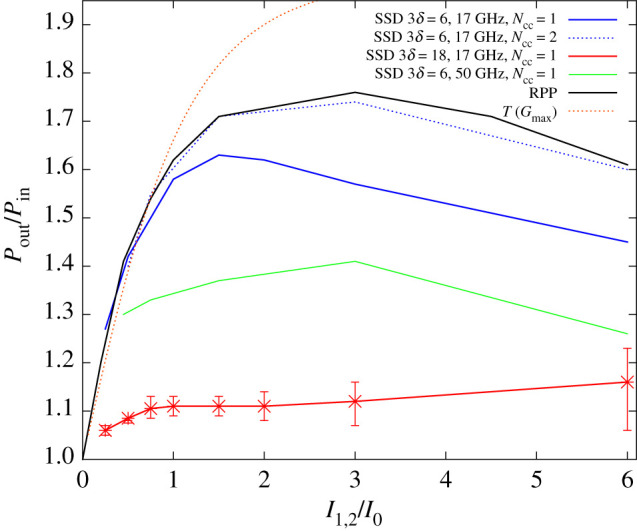
CBET power transfer relating the transmitted power of beam 2 to its incident power as a function of the incident beam intensity for the cases of RPPs (black curve) and SSD with 3*δ* = 6 (blue) as well as 18 (red) and with *ν*_mod_ = 17 GHz as well as *ν*_mod_ = 50 GHz (green curve). All solid curves correspond to a single colour cycle *N*_cc_ = 1. The dashed blue line shows the single case with two SSD colour cycles (*N*_cc_ = 2), otherwise for the same parameters as the 3*δ* = 6 and *ν*_mod_ = 17 GHz case. The dashed curve in orange corresponds to the analytic model for power transfer, *T*(*G*) as explained in §2c. (Online version in colour.)

We have also performed simulations with other laser beam intensity values, in the intensity interval 0.5 < *I*_1,2_/*I*_0_ < 6, for the three different SSD cases and for RPPs. The results of these simulations for the CBET net transfer rate *P*_out_/*P*_in_ are summarized in figure 5. Note that the values shown in this figure are based on the ensemble average over simulation results from a series of realizations for RPP beams and for SSD beams. For RPP beams the average was made over eight realizations, for the SSD cases over two realizations.

The net transfer increases in the intensity regime *I*_1_, *I*_2_ < 2*I*_0_. In ref. [15] it has already been shown that the increase of *T* = *P*_out_/*P*_in_ for RPP beams follows for *I*_1,2_ < 0.75*I*_0_ roughly the analytical model mentioned in §2c from ref. [33]. To illustrate this, the corresponding transfer *T*(*G*) for *G* = *G*_max_, is also shown in figure 5. Although this standard model for CBET does not account for speckle structure or beam self-interaction, our simulations confirm its validity up to the onset of nonlinear effects due to speckle structure. Below *I*_1,2_/*I*_0_ ≃ 0.75 the fluctuations of the transfer depend only statistically (from realization to realization) on the number of speckles in the region of crossing beams. For values above *I*_1,2_/*I*_0_ ≃ 0.75 the speckle structure in the RPP beams matters, due the onset of nonlinear processes related to beam self-interaction [11].

**Figure 6.**
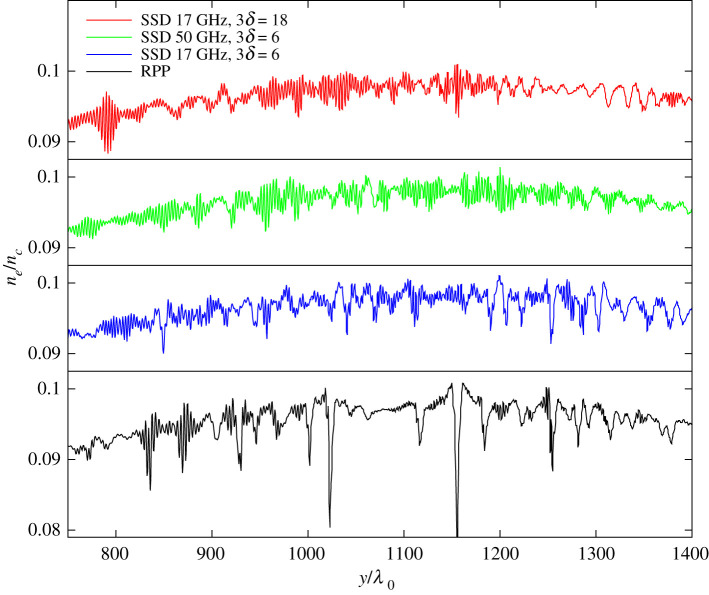
Plasma electron density lineout, *n*_*e*_/*n*_*c*_, along the *y*-axis taken at *x*/*λ*_0_ = 1600 and $t=40\tau_{\vartheta }$ for the cases of SSD 17 GHz with 3*δ* = 6 (blue) as well as 3*δ* = 18 (red), SSD 50 GHz with 3*δ* = 6 (green) and RPP (black curve). The plasma is sub-(syper-)sonic for *y*/*λ*_0_ < ( > )1100 All cases with single colour cycle *N*_cc_ = 1. (Online version in colour.)

The simulations for the case with lower bandwidth SSD, 17 GHz with 3*δ* = 6, shown in the blue line of figure 5, indicate that only the nonlinear processes in the beams can be mitigated with respect to RPPs. This is emphasized by the fact that for lower intensities up to *I*_2_/*I*_0_ ≃ 0.75, in absence of nonlinear processes, the lower bandwidth SSD case reproduces the RPP case values. The same is seen when inspecting density perturbations comparing the RPP and the SSD cases, see figure 6: the RPP case shows the strongest nonlinear structures due to the presence of speckles, which still are present for the SSD case with 17 GHz, 3*δ* = 6 SSD. The higher bandwidth cases for SSD, 17 GHz with 3*δ* = 18 and 50 GHz with 3*δ* = 6, seen in the red and green curves of figure 5, respectively, clearly diminish the CBET net transfer with respect to RPPs. This holds already for the lower intensity range in which nonlinear effects due to self-interaction in the beams do not arise. The latter is a clear signature that the incoherence of the average beams 1 and 2 induces, smaller density perturbations at the wavelength $\lambda_{0}/(2\sin\vartheta /2)$ (i.e. approx. 1 µm for the case discussed here) as expected from the coupling via the $\nabla U_{\text{cross}}$ term in equation (2.5b), ∼Γ*a*_1_*a*_2_. The amplification of beam 2 via CBET is hence reduced in time average due to temporarily incoherent coupling between the beams [34]. The SSD cases at high effective bandwidth furthermore show a relatively weak dependence of the net transfer as a function of the incident beam intensity, with, however, an increasing standard deviation, i.e. significant fluctuations between different realizations at high intensity.

As already emphasized, see figure 3, the SSD case with greater phase depth, *δ* = 18 results in systematically lower net transfer with respect to all other cases. SSD parameters in a similar range should be available to our knowledge at the French LMJ at *ν*_mod_ = 14.25 with a phase depth of 3*δ* = 15 [8]. The higher phase modulation proves to suppress not only nonlinear effects arising in speckles but also it leads to an efficient reduction of the coupling efficiency between the average beams.

## 4. Conclusions

We have studied the effects of spatio-temporal smoothing techniques on CBET for the case of two laser beams of equal frequency that cross each other in a plasma. We have considered crossing under a relatively small angle of 20^°^ in a plasma with close to sonic flow in the direction transverse to the common component of light propagation k-vector. This configuration represents an example of basic configuration for the CBET studies of relevance to many experiments. We have compared CBET with spatially smoothed laser beams via RPPs with simulation results that employ beams with spatio-temporal smoothing by SSD. SSD is the technique currently available on all large scale laser facilities concerned with problems due to CBET.

We have studied the influence of SSD on the net transfer rate of CBET, on the angular spread as well as on the plasma-induced incoherence in the transmitted laser beams. It is found that, in order to cause impact on the CBET net transfer rates, SSD needs to be introduced with sufficiently high laser bandwidth in order to be effective as compared to the plasma induced incoherence already induced in the RPP beam interactions. The plasma induced smoothing process that is responsible for the temporal incoherence inside the plasma already results in coherence times on the order of a picosecond for ICF relevant laser–plasma conditions. For beams crossing at angles $\vartheta <30^{\circ }$ the effective SSD bandwidth needs to be at least on the order of several hundreds of GHz in order to produce a considerable reduction in the CBET rates. Similar to what was observed for the stimulated backward scattering instability [35], our results show that increasing the phase depth of the SSD technique is far more efficient to control the crossed beam energy transfer rate than increasing the modulation frequency of SSD.

## Supplementary Material

Description of the Harmony code for CBET

## Data accessibility

A file with details on the model equations, the numerical scheme and the particular conditions of the Harmony code is provided in the electronic supplementary material. The code Harmony used to produce our numerical results is explained in detail in ref. [29] as well as in ref. [11] for the current configuration. The data used for figures 1–5 can be provided with gnuplot parameters for data extraction. The data volume of each file, however, exceeds several hundred MB.
