# Supplementary material for: Crossed beam energy transfer between optically smoothed laser beams in inhomogeneous plasmas
Source: Philos Trans A Math Phys Eng Sci. 2020 Oct 12;378(2184):20200038. doi: 10.1098/rsta.2020.0038 (PMC7658752; doi:10.1098/rsta.2020.0038)
Supplement: Description of the Harmony code for CBET [file rsta20200038supp1.pdf]

Harmony for Crossed beam energy transfer in the presence of laser speckle ponderomotive

S. Hüller,* G. Raj, and D. Pesme
Centre de Physique Théorique (CPHT), CNRS, Ecole Polytechnique, IP Paris, 91128 Palaiseau, France

I. INTRODUCTION

The laser-plasma configurations in ICF experiments of concern for CBET involve two laser beams with wave vectors and frequencies $(\vec{k}_1,\omega_1)$ and $(\vec{k}_2,\omega_2)$, crossing at an angle θ and leading to induced [1–3] or stimulated Brillouin scattering (SBS) of one beam into the other. The laser light beams scatter off the grating of ion acoustic waves (IAW) produced by the ponderomotive force of the two beams. In most of the experimental configurations, plasmas are inhomogeneous and are flowing with a velocity $\vec{v}_p$. Therefore, the CBET requires that the three-wave SBS resonance conditions are fulfilled for wave vectors (momentum) and frequencies (energy): $\vec{k}_s \equiv \vec{k}_1 - \vec{k}_2$ and $\omega_1-\omega_2 \equiv \omega_s + \vec{k}_s\cdot\vec{v}_p$ respectively, where ω_s and $k_s \simeq 2|k_1|\sin(\theta/2)$ are the IAW frequency and wave number for light beams crossing at the angle θ.

II. MODELING CBET BETWEEN TWO BEAMS

In the following we describe the model in our code Harmony[4] for the interaction between two laser beams crossing at the angle θ in a plasma with motion on the acoustic time scale . The particular configuration corresponds to two ‘s’-polarized beams crossing at a relatively small angle θ, and having their common wave vector component along the positive x direction; ion acoustic waves (IAWs) propagate along the y direction. The purpose is to allow the two ‘s’-polarized beams, with wave vectors and frequencies $(\vec{k}_1,\omega_1)$ and $(\vec{k}_2,\omega_2)$, to interact via IAWs that are described by a hydrodynamic model, see later. In the configuration considered for CBET, the main mechanism is Stimulated Brillouin scattering (SBS) at small scattering angles. SBS matching conditions are satisfied when $v_{p,y}(y)/c_s = (\omega_1-\omega_2-\sigma\omega_s)/(c_s k_s)$, where σ is the sign of $\omega_1-\omega_2-(\vec{k}_1-\vec{k}_2)\cdot\vec{v}_p$, and where $c_s \equiv [(c_{se}^2/(1+k_s^2\lambda_{De}^2)+3v_i^2]^{1/2}$ is the IAW velocity, with $c_{se}\equiv(ZT_e/m_i)^{1/2}$, where T_e is the electron temperature, λ_{De} the Debye length, v_i the ion thermal velocity, m_i and Z are the ion mass and charge number, respectively. In the version of Harmony for CBET, we consider the complex electric field envelope as the superposition of two beams incident at the angles $\pm\theta/2$ to the x-axis, where $\vec{k}_{j\|}$ and $\vec{k}_{j,y}$ are the parallel and transverse components of the wave vectors, respectively, with $|\vec{k}_{j\|}| = |\vec{k}_j|\cos(\theta/2)$, $|\vec{k}_{j,y}| = |\vec{k}_j|\sin(\theta/2)$ and $|\vec{k}_j| = (\omega_j^2-\omega_p^2)^{1/2}/c$ (j=1,2). The electric field can then be written as

$$E(\vec{x},t) = \frac{\hat{E}}{2}e^{i\vec{k}_{1\|}\cdot\vec{x}}\left[a_1 e^{-i\omega_1 t+i\vec{k}_{1,y}\cdot\vec{y}} + a_2 e^{-i\omega_2 t+i\vec{k}_{2,y}\cdot\vec{y}}\right]+cc, \quad (1)$$

where a_1, and a_2 are the electric field amplitudes of both beams normalized to the field strength $\hat{E}$. For not too large angles θ between the two beams, the paraxial approximation can be used, and for $|\omega_1-\omega_2|\equiv 0$ and $\vec{k}_{2,y} = -\vec{k}_{1,y}$ the propagation of the incoming beams can be described by paraxial equations for $a(\vec{x},t)\equiv a_1\exp\{i\vec{k}_{1,y}\cdot\vec{y}\} + a_2\exp\{-i\vec{k}_{1,y}\cdot\vec{y}\}$, or, alternatively, individually for a_1, a_2, coupled to the plasma density perturbations[5]

$$\left[2i\omega_1(\partial_t + v_{gx}\partial_x) + c^2(\nabla^2)_\perp\right]a(\vec{x},t) = \omega_p^2\frac{\delta n}{n_e}a(\vec{x},t)\ , \quad (2)$$

where $v_{gx}\equiv v_g\cos(\theta/2)$, $\omega_p = (n_e e^2/\varepsilon_0 m_e)^{1/2}$ is the electron plasma frequency; $n_c = \varepsilon_0 m_e\omega_0^2/e^2$ denotes the critical density, m_e and e being the plasma electron mass and charge respectively, $\delta n = (n-n_e)$ is the density perturbation about the equilibrium density n_e.

Eq. (2) describes the evolution of the electromagnetic wave amplitudes in the paraxial approximation on the scale of hydrodynamical evolution and long wavelength IAW response of $\delta n/n_e$. The high-frequency response for IAWs due to backscattered SBS is treated in Harmony via a harmonic decomposition[4], that one has to consider for large angles $\theta \sim 180^o$.

In Harmony Eq. (2) is solved via a standard paraxial wave solver. It is based on a Crank-Nicolson scheme in the configuration space x,y and a simple time retardation for the forward propagation part, $(\partial_t + v_{gx}\partial_x)$. The resulting tri-diagonal matrix system is then solved in a straight-forward manner by upstream-downstream recursion on the regular spatial grid in y-direction. Time-dependent boundary conditions are imposed at $x=0$ for $a(x=0,y,t)$. The spatial system is chosen such that the light fields have exiting solutions at the rear, $x=L_x$ and its values are negligible at the lateral boundaries.

The plasma dynamics is described by the standard hydrodynamic equations in the isothermal approximation,

$$\partial_t n + \nabla\cdot(n\vec{v}) = 0, \quad (3a)$$

$$[\partial_t + \vec{v}\cdot\nabla]\,\vec{v} + c_s^2\frac{\nabla n}{n} + 2\nu_s\vec{v} = -c_{se}^2\frac{\nabla U}{T_e}, \quad (3b)$$

where U stands for the ponderomotive potential and ν_s for the ion acoustic damping. In our simulations we assume for ν_s a linear wave-number dependence, and $n\nu_s\vec{v}$ is computed in Fourier space with $\nu_s(k_s) = \hat{\nu}\ \omega_s(k_s)$, accounting for Landau damping[4, 5].

The system of Eqs. (3a-3b) is solved in using the Eulerian hydrodynamic “Clawpack” wave solver package, here in two spatial dimensions, developed by R. J. Le Vecque, see http://www.amath.washington.edu/ claw/; J. Comp. Phys. **131**, 327 (1997). The isothermal module of this wave solver package can be adapted such that the coupling to the electromagnetic fields, as well as the wave damping term, enter as an explicite source term to the momentum equation, Eq. (3b).

For the electric field resulting from the superposition of two beams of equal frequency, $a(\vec{x},t)\equiv a_1\exp\{i\vec{k}_{1,y}\cdot\vec{y}\} +$

* stefan.hueller@polytechnique.edu

$a_2 \exp\{-i\vec{k}_{1,y}\cdot\vec{y}\}$, the ponderomotive force $\nabla U \propto \nabla|a(\vec{x},t)|^2$ can be subdivided into two separate contributions, provided that the central wave vector component in y for each beam, $|\vec{k}_{1,y}|$ and $|\vec{k}_{2,y}|$ exceeds the wave number spread $\triangle k$ related to the angular aperture of each RPP beam; the latter is a function of the focusing f-number, namely[6] $\triangle k \equiv |\vec{k}_1|/[1+4f^2]^{1/2} \sim |k_1|/(2f)$.

Keeping in mind the condition, that for two separate fields the wave number separation needs to be greater than the angular aperture of the fields, $|\vec{k}_{2,y}-\vec{k}_{1,y}| > \triangle k$, the ponderomotive force in the right-hand side of the equation of motion can be expressed in two distinct terms namely $\nabla U = T_e\Gamma\nabla|a(\vec{x},t)|^2 \equiv \nabla U_{cross} + \nabla U_{self}$, given by $\nabla U_{cross}/T_e = \Gamma\,\nabla\left(a_1 a_2^* e^{2i|\vec{k}_1|y\sin(\theta/2)} + cc.\right)$, and $\nabla U_{self}/T_e = \Gamma\ \nabla(|a_1|^2+|a_2|^2)$, with the coupling coefficient $\Gamma = \langle v_{\rm osc}^2\rangle/v_{\rm th}^2 = v_{\rm osc}^2/(2v_{\rm th}^2)$ which involves the thermal velocity $v_{\rm th} = (T_e/m_e)^{1/2}$ and the electron quiver velocity $v_{\rm osc} = e\hat{E}/(m_e\omega)$ of the field $\hat{E}$ to which a_1 and a_2 are normalized. The ponderomotive force contribution ∇U_{cross} acts essentially on the plasma fluid due to the beating between the two waves a_1 and a_2 in SBS. The ponderomotive force contribution denoted by ∇U_{self} is accounting for ponderomotive self- interaction in the paraxial approximation for each individual beam. This self-interaction can be associated with self-focusing and with forward-SBS inside each beam. Note, that in contrast to the case of large angles, both contributions to the ponderomotive force have major components along the y-direction, i.e. across the main common propagation axis x.

III. THE SIMULATION CONFIGURATION IN HARMONY

In the two dimensional (2D) simulations with our code HARMONY [4, 5], we have chosen a crossing angle of θ =20°. The plasma flow profile follows a linear ramp in y-direction, as defined by $v_{p,y}(y)/c_s = (y-L_y/2+L_v)/L_v$ with L_v (=200λ_0 in the simulations) as the gradient length, so that, assuming equal frequencies for both beams, $\omega_1=\omega_2=\omega_0$, SBS matching occurs at $y = L_y/2$ in the center. We have performed simulations for the case when both entering beams have the same average intensity, $I_{02}=I_{01}$. For this case, $I_{02}/I_0 = I_{01}/I_0 \equiv 1$, the reference intensity, I_0, corresponds for λ_0 =0.35μm light to an average laser intensity of $I_0 = I_L \simeq 0.9\times10^{15}$W/cm^2 at T_e =3keV.

The laser wave amplitude, $a(\vec{x},t)$ in Eq.(2), at the laser entrance boundary $x=0$ is generated via a Fourier series for two separate wave-fields for a_1 and a_2. In Fourier space, each of the wave-field contributions is centered around the wave vector components $|\vec{k}_{1,y}| = -|\vec{k}_1|\sin(\theta/2)$ and $|\vec{k}_{2,y}| = |\vec{k}_1|\sin(\theta/2)$, respectively. For RPP with $i = 1\ldots N_{\rm RPP}$ elements in each beam, with random phases $\phi_{j,i}$, this reads

$$a_j(x=0,y) = e^{i\vec{k}_{j,y}\cdot\vec{y}} \sum_{k_i=-\triangle k}^{\triangle k} |\hat{a}_{j,i}| e^{ik_i y + i\phi_{j,i}},\ j=1,2\ , \qquad (4)$$

where the i-th element has the wave number k_i and amplitude $\hat{a}_{j,i}$, with the spacing between them, $k_{i+1}-k_i = 2\,\triangle k/N_{\rm RPP}$. The width in k_y, is given by[6] $\triangle k \equiv |\vec{k}_1|/[1+4f^2]^{1/2} \sim k_1/2f$. The total field composed of two beams must have zero elements in the Fourier series for $|k_y| < |\vec{k}_{j,y}| - \triangle k$, corresponding to the angular interval around $\theta = 0^o$. For an angular separation of the beams the condition $\triangle k < |\vec{k}_{1,y}|$ has to be fulfilled.

For Smoothing via Spectral Dispersion, SSD, the procedure is similar to the one in Eq. 4, except that total phase consists of the random phase for RPP $\phi_{j,i}$ and the SSD phase from a deterministic expression, i.e. $\phi_{j,i} = \phi_{j,i,\rm RPP} + \phi_{j,i,\rm SSD}$, with $\phi_{j,i,\rm SSD} = 3\delta\sin[2\pi\nu_{\rm mod}t + \pi N_{\rm cc}(k_i/\triangle k)]$ in which $N_{\rm cc}$ indicates the number of so-called colour cycles of SSD (typically $N_{\rm cc}$ =1, 2, 4, 8. The phase modulation depends hence on the modulation frequency $\nu_{\rm mod}$ and the phase depth δ; the latter generally depends on the laser frequency, i.e. for frequency tripled laser light at λ_0 =0.35μm, a three times higher value has, 3δ to be taken.

We have performed simulations with a single field array a and with separate field arrays a_1 and a_2. We have verified that the simulations of both types of configurations show the same results when both terms ∇U_{cross} and ∇U_{self} and taken into account in the configuration with two field arrays.

For all simulations we have chosen a domain of 4500λ_0 in length and 2300λ_0 in width, i.e. along the x and the y axes, respectively, with a resolution of 4500 grid points in x, and 4096 grid points in y. The beams have a common wave vector component along x and opposite wave vector components along y. In this chosen geometry, the gradients of the plasma profiles in density and velocity point predominantly along the y-direction. The density profile is parabolic around the center, $y = L_y/2$, given by $n_e(y)$ =0.1n_c $\exp-[(y-L_y/2)/1615\lambda_0]^2$. We apply a linear density ramp starting at x =0 over 500λ_0 along x in order to avoid boundary effects at the laser entry and a linear flow ramp with sonic flow in the center of the crossing beams, $v_{p,y}(y=L_y/2) = +c_s$, and a flow gradient L_v =200λ_0. We have focused our study on the case when both beams have equal intensity I_{01}=I_{02} at the entrance $x=0$. Both beams have the same focusing $f-$number, namely f =6 for RPP beams. The coefficient $\hat{\nu}$ takes the value $\hat{\nu}$ =0.1.

[1] W. L. Kruer, S. C. Wilks, B. B. Afeyan, and R. K. Kirkwood, Phys. Plasmas **3**, 382 (1996).

[2] R. K. Kirkwood, B. B. Afeyan, W. L. Kruer, B. J. MacGowan, J. D. Moody, D. S. Montgomery, D. M. Pennington, T. L. Weiland, and S. C. Wilks, Phys. Rev. Lett. **76**, 2065 (1996).

[3] V. V. Eliseev, W. Rozmus, V. T. Tikhonchuk, and C. E. Capjack, Physics of Plasmas **3**, 2215 (1996).

[4] S. Hüller, P. E. Masson-Laborde, D. Pesme, M. Casanova, F. Detering, and A. Maximov, Phys. Plasmas **13**, 022703 (2006).

[5] D. Pesme, S. Hüller, J. Myatt, C. Riconda, A. Maximov, V. T. Tikhonchuk, C. Labaune, J. Fuchs, S. Depierreux, and H. A. Baldis, Plasma Phys. Controlled Fusion **44**, B53 (2002).

[6] H. A. Rose and D. F. DuBois, Phys. Fluids B **5**, 3337 (1993).
